# The synergistic effects of mechanical ventilation and intrauterine inflammation on cerebral inflammation in preterm fetal sheep

**DOI:** 10.3389/fncel.2024.1397658

**Published:** 2024-06-19

**Authors:** Nhi T. Tran, Ainsley Somers, Kayla Vidinopoulos, Zahrah Azman, Yen Pham, Valerie A. Zahra, Kyra Y. Y. Chan, Stuart Hooper, Kelly Crossley, Beth J. Allison, Robert Galinsky, Graeme R. Polglase

**Affiliations:** ^1^The Ritchie Centre, Hudson Institute of Medical Research, Clayton, VIC, Australia; ^2^Department of Obstetrics and Gynaecology, Monash University, Clayton, VIC, Australia; ^3^Department of Paediatrics, Monash University, Clayton, VIC, Australia

**Keywords:** intrauterine inflammation, chorioamnionitis, preterm brain, ventilation, neuroinflammation

## Abstract

**Background:**

Intrauterine inflammation and the requirement for mechanical ventilation independently increase the risk of perinatal brain injury and adverse neurodevelopmental outcomes. We aimed to investigate the effects of mechanical ventilation for 24 h, with and without prior exposure to intrauterine inflammation, on markers of brain inflammation and injury in the preterm sheep brain.

**Methods:**

Chronically instrumented fetal sheep at ~115 days of gestation were randomly allocated to receive a single intratracheal dose of 1 mg lipopolysaccharide (LPS) or isovolumetric saline, then further randomly allocated 1 h after to receive mechanical ventilation with room air or no mechanical ventilation (unventilated control + saline [UVC, *n* = 7]; *in utero* mechanical ventilation + saline [VENT, *n* = 8], unventilated control + intratracheal LPS [UVC + LPS, *n* = 7]; *in utero* ventilation + intratracheal LPS [VENT + LPS, *n* = 7]). Serial fetal blood and plasma samples were collected throughout the experimental protocol for assessment of blood biochemistry and plasma interleukin (IL)-6 levels. After 24 h of mechanical ventilation, fetal brains were collected for RT-qPCR and immunohistochemical analyses.

**Results:**

LPS exposure increased numbers of microglia and upregulated pro-inflammatory related genes within the cortical gray matter (GM) and subcortical white matter (SCWM) (*p_LPS_* < 0.05). Mechanical ventilation alone increased astrocytic cell density in the periventricular white matter (PVWM) (*p_VENT_* = 0.03) but had no effect on pro-inflammatory gene expression. The combination of ventilation and LPS increased plasma IL-6 levels (*p* < 0.02 vs. UVC and VENT groups), and exacerbated expression of pro-inflammatory-related genes (*IL1β*, *TLR4*, *PTGS2*, *CXCL10*) and microglial density (*p* < 0.05 vs. VENT).

**Conclusion:**

This study demonstrates that 24 h of mechanical ventilation after exposure to intrauterine inflammation increased markers of systemic and brain inflammation and led to the upregulation of pro-inflammatory genes in the white matter. We conclude that 24 h of mechanical ventilation following intrauterine inflammation may precondition the preterm brain toward being more susceptible to inflammation-induced injury.

## 1 Introduction

Preterm infants have an increased risk of brain injury which underlies adverse neurodevelopmental outcomes. The causes of preterm brain injury are multifactorial, and most likely due to prenatal and postnatal compromise on top of implicit immaturity (Galinsky et al., 2018). Many preterm infants require respiratory support to assist with gas-exchange after birth. Whilst life-saving, there is now substantial preclinical and clinical evidence that prolonged mechanical ventilation of premature infants induces brain injury, termed ventilation-induced brain injury (VIBI) (Serenius et al., 2004; Aly et al., 2012; Barton et al., 2015; de Medeiros et al., 2022).

The initiation of mechanical ventilation can cause VIBI through two distinct pathways; (1) the haemodynamic pathway, whereby changes in intrapulmonary pressures causes pulmonary hemodynamic instability, alters cardiac output (Shekerdemian and Bohn, 1999; Polglase et al., 2005, 2009) and, as a consequence, causes fluctuations to cerebral blood pressure and flow (Hillman et al., 2010; Polglase et al., 2012) and (2) the initiation of a pulmonary inflammatory response that migrates systemically and then to the central nervous system (Barton et al., 2015; Vidinopoulos et al., 2023). This neuroinflammatory response involves gliosis (proliferation and activation of microglia and astrocytes) in the periventricular and intragyral white matter (Barton et al., 2015, 2016; Stojanovska et al., 2018; Chan et al., 2022). The activation of cerebral glia and pro-inflammatory mediators have putative roles in preterm newborn brain injury (Khwaja and Volpe, 2007; Kelly et al., 2023); as such, neuroinflammatory mechanisms are believed to play a central role in VIBI pathology.

A major antecedent of preterm birth is exposure to intrauterine inflammation, which can manifest clinically as chorioamnionitis (Romero et al., 2006; Galinsky et al., 2013). In response to intrauterine inflammation, the fetus mounts a fetal inflammatory response, resulting in upregulation of pulmonary, systemic and cerebral inflammatory mechanisms. As a consequence, preterm infants have reduced respiratory function and oxygenation at birth, increased need for intubation and positive pressure ventilation (PPV) (Malaeb and Dammann, 2009; Villamor-Martinez et al., 2019; Panneflek et al., 2023), and an increased risk of brain injury and adverse neurodevelopmental outcomes including cerebral palsy (Galinsky et al., 2018). Given that intrauterine inflammation and mechanical ventilation can independently trigger neuroinflammatory responses, there is a potential for a “double-hit” to the immature brain if PPV is initiated after exposure to intrauterine inflammation.

This study aimed to determine: (1) The effects of 24 h of *in utero* mechanical ventilation on markers of inflammation and injury to the preterm brain, and (2) whether exposure to intrauterine inflammation, induced by intra-tracheal administration of lipopolysaccharide (LPS), amplifies markers of neuroinflammation and injury. We hypothesized that exposure to intrauterine inflammation would augment the ventilation-induced increase in gene and cellular markers of neuroinflammation and injury in preterm fetal sheep.

## 2 Materials and methods

### 2.1 Animal ethics and welfare

All experiments were performed in accordance with the ARRIVE guidelines 2.0 (Supplementary File 1; Percie du Sert et al., 2020). The use of animals was approved by Monash Medical Centre Animal Ethics Committee (MMCA/2020/15) and was conducted in accordance with the Australian Code of Practice for the care and use of Animals for Scientific Purposes established by the National Health and Medical Research Council of Australia.

### 2.2 Sterile animal surgery

Pregnant Border-Leicester ewes carrying singletons or twins were utilized in this study. At 110 days of gestation (dGA; term is ~148 dGA), after withdrawal of food for at least 18 h, ewes were anaesthetized with intravenous sodium thiopentone (20 mg·kg^−1^) and intubated. General anesthesia was maintained by inhalation of 1.5–3.5% isoflurane in oxygen. Under sterile conditions, a midline laparotomy was performed on pregnant ewes to expose the fetal head, neck and left forelimb. In the case of a twin pregnancy, only one fetus was operated on. Instrumentation of pregnant ewes and their fetuses has been previously described in Vidinopoulos et al. (2023). Briefly, a tracheostomy was performed to secure a modified reinforced endotracheal tube in the lower trachea and was connected to a saline filled large-bore ventilation tube. A separate saline-filled catheter was inserted into the upper trachea and connected to the ventilation tube externally to create an exteriorised tracheal loop, to allow normal flow of lung liquid. The left brachial artery and left jugular vein were catheterised for serial arterial blood sampling and antibiotic administration, respectively. The fetus was returned to the uterus and the catheters and tracheal loop were exteriorised via the ewe’s right flank. Postoperative analgesia was maintained for 3 days via a transdermal fentanyl patch on the left hind leg of the ewe (75 μg·h^−1^; Jansen Cilag, North Ryde, NSW, Australia). Antibiotics were administered i.v. to the ewe (ampicillin, 800 mg and engemycin, 500 mg) and the fetus (ampicillin, 200 mg) for 3 consecutive days after surgery. Three to 5 days of post-operative recovery were allowed prior to commencing the experiment.

### 2.3 Lipopolysaccharide administration and *in utero* ventilation

At 113–115 dGA, fetuses were randomly assigned to four groups: unventilated controls without (UVC; *n* = 7) or with intratracheal LPS (UVC + LPS; *n* = 7); or ventilated controls with (VENT + LPS; *n* = 7) or without intratracheal LPS (VENT; *n* = 8). Ventilation tubing was disconnected, and lung liquid passively drained. To induce localized acute inflammation, LPS (*Escherichia coli*, 055:B5, Millipore Sigma, MO, USA; 1 mg in 2 mL saline) was infused into the tracheal tube 1 h before the onset of *in utero* ventilation (Polglase et al., 2009). For UVC and VENT fetuses, 2 mL of saline was administered. After 1 h, the endotracheal tube was connected to a neonatal ventilator (Babylog 8000+, Dräger, Lübeck, Germany) and ventilated (Vidinopoulos et al., 2023). Both VENT and VENT + LPS fetuses were ventilated *in utero* with non-humidified air with a peak inspiratory pressure (PIP) 25–40 cmH_2_O targeting a tidal volume (*V*_T_) between 3 and 5 mL·kg^−1^, positive end expiratory pressure (PEEP) 4 cmH_2_O, flow 10 L·min^−1^, 60 inflations·min^−1^ and FiO_2_ 21% for 24 h. The *in utero* ventilation technique in fetal sheep is an established model to investigate the mechanisms of ventilation-induced injury independent of haemodynamic instability involved with the cardiorespiratory transition and removes potential confounders that occur when ventilating preterm neonates for extended periods of time *ex utero*, including: maintaining oxygen, cardiovascular support, nutrition, temperature and corticosteroid exposure (Blanco et al., 1987; Allison et al., 2008). Further, it enables the mechanical ventilation of preterm fetal sheep at a much younger gestation than what would be viable *ex utero*, allowing us to investigate the sheep brain that is comparable to a preterm infant (Back et al., 2006).

Fetal arterial blood was sampled for blood gas measurements (ABL80 FLEX, Radiometer Medical ApS, Denmark) before LPS/vehicle infusion (Pre-LPS), before ventilation (Pre-Vent), +15, +30, +45, +60 min post-vent and +3, +6, +9, +12 h, +24 h post-vent. Pre-vent, +3, +6 and, +12 h post-vent plasma samples were collected for IL-6 analysis. At 24 h, the ewe and fetus were humanely euthanised with an intravenous overdose of sodium pentobarbitone (100 mg·kg^−1^ i.v.; Valabarb Euthanasia Solution; Jurox, NSW, Australia) via the maternal jugular vein catheter. UVC fetuses were instrumented and received intratracheal vehicle/LPS but were not ventilated and were euthanised at the same age as the VENT groups. At post-mortem, the fetal brain was removed, weighed and hemisected. The left hemisphere was dissected coronally to obtain a ~1 cm block at the level of the ansate sulcus. The subcortical white matter (SCWM) was collected via microdissection of the white matter within the 1st and 2nd gyri, samples were pooled and immediately snap frozen in liquid nitrogen. Similarly, cortical gray matter (GM) of the 1st and 2nd gyri were pooled and immediately snap frozen in liquid nitrogen. The periventricular white matter (PVWM) was located as the white matter surrounding the ventricle above the subventricular zone, tissue was microdissected, collected and snap frozen. All collected tissue was stored at −80°C for RT-qPCR analysis. The right hemisphere was immersion fixed in 10% neutral-buffered formalin for immunohistochemical analyses.

### 2.4 Plasma IL-6 analysis

Arterial blood was collected via the fetal brachial artery catheter before ventilation (Pre-Vent), and at 3, 6, and 12 h for assessment of plasma IL-6 using a sandwich enzyme-linked immunosorbent assay (ELISA) assay as described previously (Galinsky et al., 2020). Briefly, plates were read on a SpectraMax i3 microplate reader (Molecular Devices, CA, USA) at 450 nm to determine optical density. Standards (recombinant ovine IL-6; Kingfisher Biotech, MN, USA) were included and a standard curve was generated for every ELISA plate used (*R*^2^ > 0.99). Due to a freezer malfunction, some plasma samples could not be assessed. The final group numbers for plasma IL-6 analysis were as follows, UVC, *n* = 6; UVC + LPS, *n* = 7; VENT, *n* = 6; and VENT + LPS, *n* = 5.

### 2.5 RT-qPCR

RNA extraction, cDNA preparation and analysis were conducted as described previously (Tran et al., 2023). Briefly, RNA was extracted from frozen brain tissue (20–30 mg) using an RNA extraction kit (RNeasy Mini Kit, Qiagen, Germany) following the manufacturer’s instructions. RNA yield was determined by spectrophotometry (Nanodrop, Analytical Technologies, Biolab). cDNA was transcribed from RNA, then pre-amplified at 50 ng·μL^−1^ (SuperScript^®^ III First-Strand Synthesis System for RT-PCR kit; Invitrogen). Gene expression was analyzed using a Fluidigm Dynamic array Biomark HD system (Fluidigm, USA). Gene expression of 8 genes of interest (Table 1) was determined by relative expression calculated by change in cycle threshold (ΔCt) between each gene of interest and the geometric average of two endogenous housekeeping genes, *YWHAZ* and *RPS18*. These genes were selected based on markers identified form previous clinical and preclinical studies investigating inflammatory markers in the context of intrauterine inflammation and mechanical ventilation (Bohrer et al., 2010; Polglase et al., 2012; Barton et al., 2016; Stojanovska et al., 2022). Levels of mRNA expression relative to geometrical average of house-keeping genes were determined using the 2^−ΔΔCT^ method (Livak and Schmittgen, 2001) and expressed relative to the UVC group.

**Table 1:** Genes of interest.

Biological process	Gene name	ID	Taqman code
Inflammation	Interleukin 1 beta	*IL1B*	Oa04656322_m1
	Interleukin 6	*IL6*	Oa04656315_m1
	Interleukin 10	*IL10*	Oa03212724_m1
	Tumor necrosis factor alpha	*TNF*	Oa04655425_g1
	Toll-like receptor 4	*TLR4*	Oa04656419_m1
	Prostaglandin-endoperoxide synthase (Cyclooxygenase 2)	*PTGS2*	Oa04657348_g1
	C-X-C Motif Chemokine Ligand 2	*CXCL2*	Oa04677078_m1
	Chemokine interferon-γ inducible protein 10 kDa	*CXCL10*	Oa04655787_g1
Housekeeping genes	14–3-3 protein zeta	*YWHAZ*	Oa04913608_m1
	Ribosomal Protein S18	*RPS18*	Oa4906333_g1

### 2.6 Immunohistochemistry

**Figure 1:**
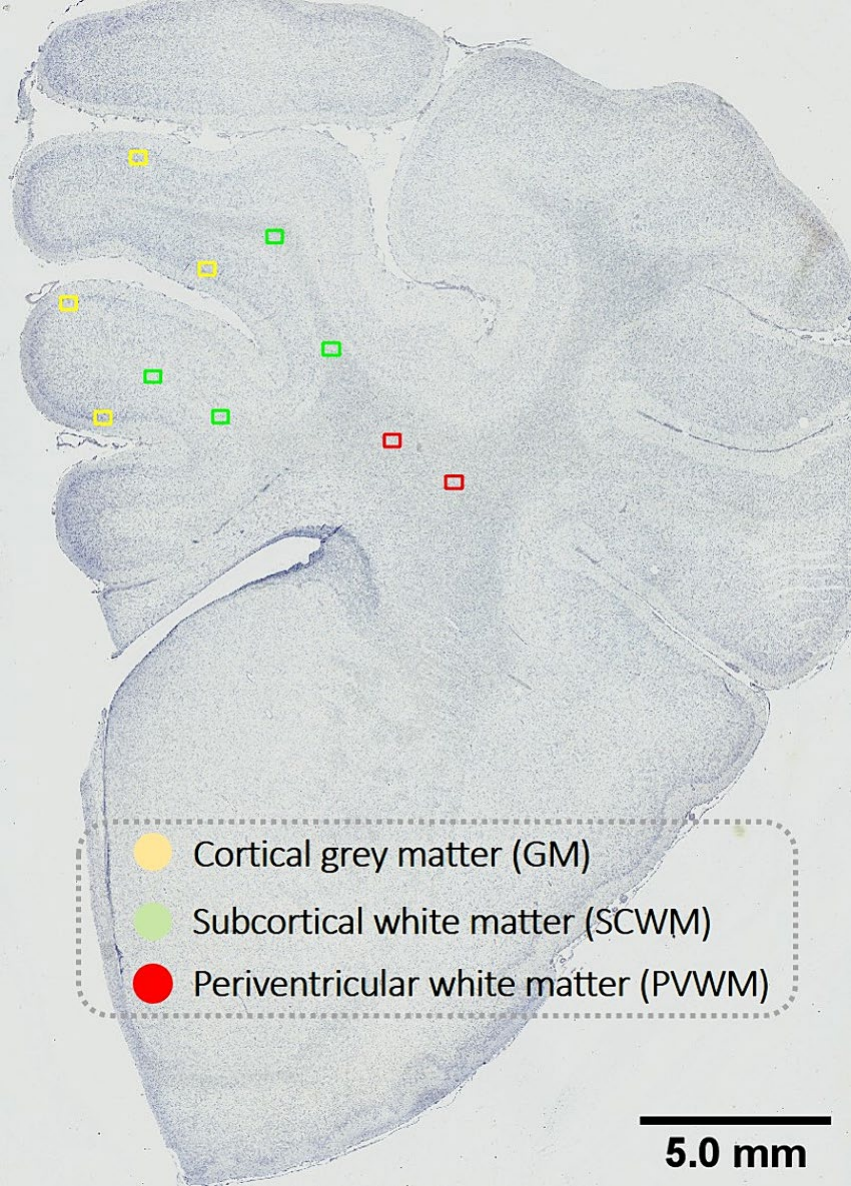
Schematic indicating fields sampled for histological assessment. Representative image for histological assessment of fields of views (FOV) sampled within the cortical gray matter (GM; yellow) and subcortical white matter (SCWM; green) regions within the first and second parasagittal gyri and the periventricular white matter (PVWM; red). Scale bar is 5 mm.

Immersion-fixed fetal brains were paraffin embedded and then microtome sectioned at 8 μm corresponding to section 720 according to the Michigan State University Sheep Atlas (Johnson et al., 2013; Figure 1). Two sections per animal per antibody were utilized. Immunohistochemical staining was conducted using primary antibodies: rabbit anti-neuronal nuclei (NeuN; 1:200; Abcam, UK; CAT#: 177487) for mature neurons; rabbit anti-ionized calcium binding adaptor molecule 1 (Iba-1; 1:250; Abcam, UK; CAT#: ab178846) for microglia; rabbit anti-glial fibrillary acidic protein (GFAP; 1:200; Abcam, UK; CAT#: ab68428) for astrocytes; rabbit anti-Olig-2 (1:200; Abcam, UK; CAT#:ab109189) for oligodendrocytes. Immunohistochemical staining protocol was conducted as described previously (Tran et al., 2023). Briefly, tissue sections were subjected to antigen retrieval using citrate buffer (10 mM Tri-sodium citrate in dH_2_O, pH 6.0; Sigma Aldrich), PBS washes, endogenous peroxidase blocking, and overnight incubation in primary antibodies at 4°C in primary diluent of 3% normal goat serum in PBS. Sections were incubated in secondary goat biotinylated anti-rabbit IgG for 2 h (1:200; Vector Laboratories, UK; CAT#: BA-100) then incubated with avidin-biotin complex (ABC Elite kit; 1:1:200 in PBS; Vectastain^®^, Vector Laboratories, UK) and visualized with 3,3′-diamniobenzidine solution (DAB; MP Biomedicals, Australia).

Prior to analyses, all slides were coded, and assessors (NTT, AS) were blinded to the treatment group. Slides were scanned at 40× magnification using Aperio Scanscope AT Turbo (Leica Biosystems, Germany). Regions of interest included the cortical gray matter (GM) and subcortical white matter (SCWM) of the first and second parasagittal gyri (i.e., GM 1st and 2nd gyri, SCWM 1st and 2nd gyri), and the periventricular white matter (PVWM) (Figure 1). For each region, 2 fields of view (FOV; 410 μm × 320 μm) were analyzed with FOV placement kept consistent across all antibodies. All analyses were averaged across a total of four FOV (two sections per subject with two FOV per region).

Total cell density for all antibodies was conducted and expressed as cells/field. For analysis of total cell density, cells were identified by their immunopositive densely stained round somas with diameters >6 μm. Specifically for Iba-1 analysis, total cell density was manually counted as cell bodies with immunopositive staining irrespective of the morphology of the processes, and ameboid microglia were identified by characteristic enlarged and round, densely stained soma with resorbed processes (Kettenmann et al., 2011). To assess area coverage of GFAP immunostaining, expressed as % area coverage, each FOV was exported from Aperio and the image was processed using ImageJ software (version 2.0.0-rc-69/1.52p, National Institutes of Health) and an optimized set threshold was used to calculate area coverage for all images using ImageJ. The optimized set threshold was conducted by calculating an average threshold range of 20 randomly selected FOV that would allow optimal detection of positive staining. The optimized threshold was then set for all slide assessments and the ‘area fraction tool’ in ImageJ was used to automatically quantify the percentage of positive immunostaining in each FOV. Microglial aggregations were also identified as dense clusters of positive staining and the aggregated area expressed as a percentage of the total brain regions of interest (SCWM, GM, PVWM).

### 2.7 Statistical analysis

All statistical analyses were conducted using GraphPad Prism (version 10.1.0; GraphPad Software, CA, United States). Data were assessed for normality using the Shapiro–Wilk Test. Data for animal characteristics and immunohistochemical were assessed using a parametric two-way ANOVA and assessed for the main effects of LPS (*p_LPS_*), ventilation (*p_VENT_*) and interactions between LPS and ventilation (*p_LPS X VENT_*). Post-hoc analysis of significant interactions and group effects was used to determine differences between groups using a Tukey’s multiple comparisons test. For mRNA expression analysis, data was non-parametric and was log10-transformed to conduct two-way ANOVA analyses as above. For plasma IL-6 and blood chemistry measurements, a parametric three-way ANOVA was used to assess the main effects of LPS (*p_LPS_*), ventilation (*p_VENT_*) and time (*p_TIME_*). Post-hoc analysis was performed on significant interactions using Tukey’s multiple comparison test or a Bonferroni’s multiple comparisons test where a significant main effect was observed. Data are presented as mean ± SD. A *p* < 0.05 was considered statistically significant for all analyses.

## 3 Results

### 3.1 Fetal characteristics, blood gas and metabolite measurements

Fetal characteristics at post-mortem (112–119 dGA) of the four groups are presented in Supplementary Table 1. LPS fetuses were older and had higher body weights than non-LPS fetuses (*p_LPS_ =* 0.024). There were no differences in male to female ratios. Due to the small numbers for each group, sex differences could not be assessed.

**Figure 2:**
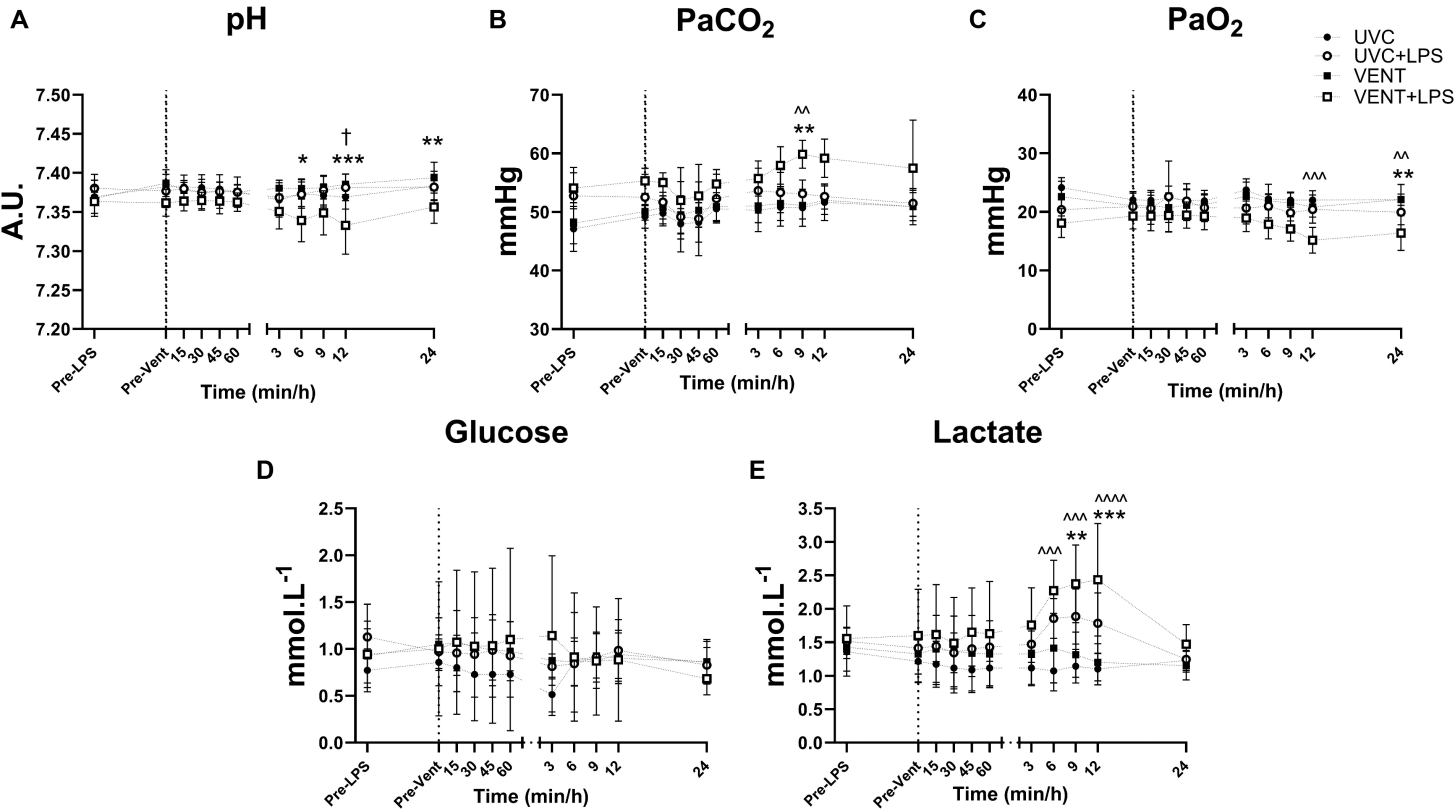
Blood gas and Metabolite measurements. Blood gas measurements of fetal arterial **(A)** pH, **(B)** partial pressure CO_2_ (PaCO_2_), **(C)** partial pressure O_2_ (PaO_2_), **(D)** blood glucose and **(E)** lactate taken pre-LPS and pre-ventilation (dotted line). Time shown relative to ventilation in min and then h. Data are mean ± SD. Unventilated control (UVC; *n* = 7), LPS unventilated (UVC + LPS; *n* = 7), ventilated vehicle (VENT; *n* = 8), and ventilated LPS (VENT + LPS; *n* = 7) fetuses. Two-way ANOVA and Tukey’s multiple comparisons. Significant differences between UVC vs. VENT + LPS indicated as *^^^p* < 0.05; *^^^^p* < 0.01; *^^^^^p* < 0.001; VENT vs. VENT + LPS indicated as *p < 0.05, **p < 0.01, ***p < 0.001; UVC + LPS vs. VENT + LPS indicated as ^†^*p* < 0.05.

Between 6 and 24 h of ventilation, arterial pH and PaO_2_ decreased and PaCO_2_ and lactate increased in VENT + LPS fetuses compared to both UVC and VENT fetuses (Figures 2A–E and Supplementary Table 2). Arterial pH was significantly lower in VENT + LPS fetuses at 12 h compared to UVC + LPS fetuses (*p* = 0.011; Figure 2A).

### 3.2 Plasma IL-6 levels

**Figure 3:**
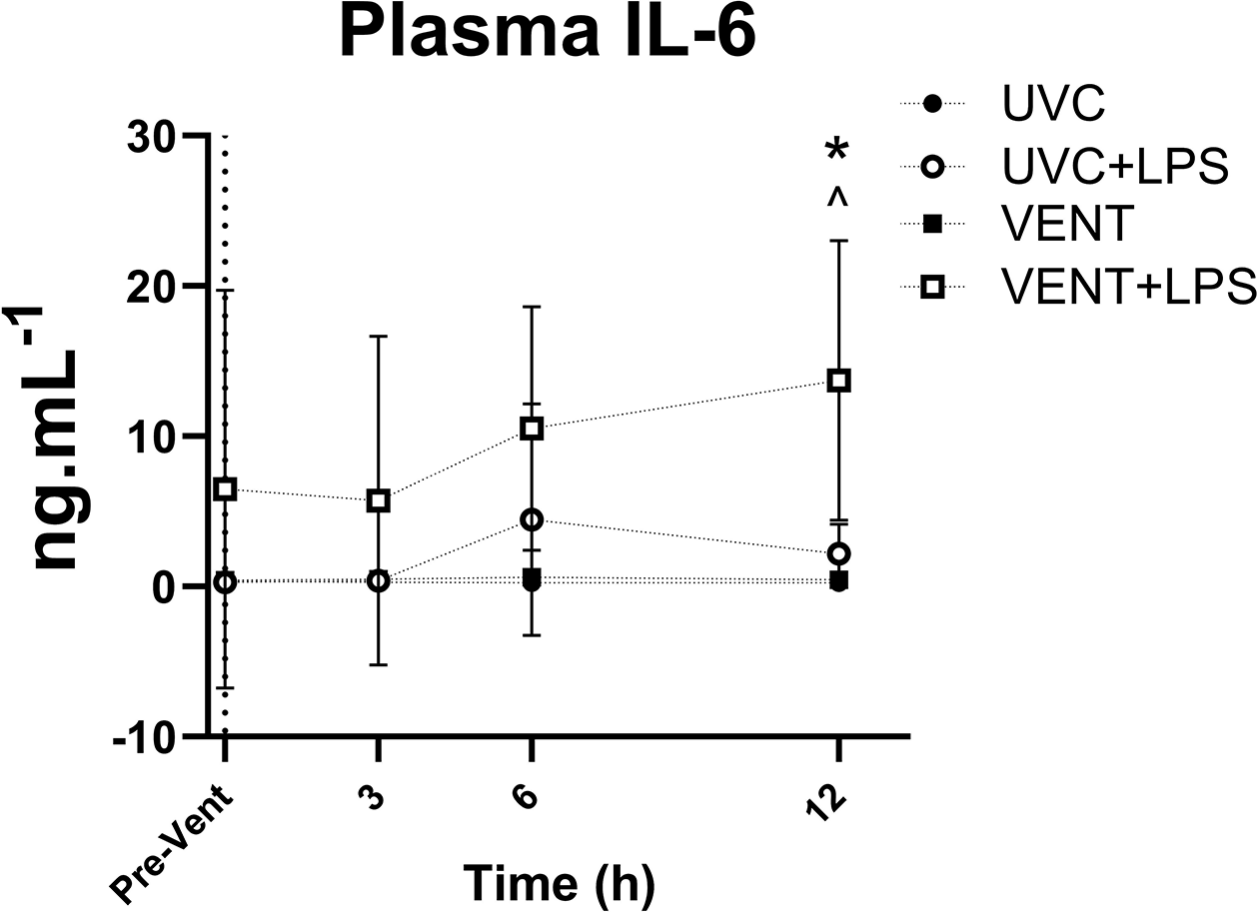
Plasma IL-6 levels. Plasma IL-6 levels taken at baseline prior to LPS and ventilation (dotted line). Time shown relative to ventilation in h. Data are mean ± SD. Unventilated control (UVC; *n* = 7), LPS unventilated (UVC + LPS; *n* = 7), ventilated (VENT; *n* = 8), and ventilated LPS (VENT + LPS; *n* = 7) fetuses. Two-way ANOVA and Tukey’s multiple comparisons. Significant differences between UVC vs. VENT + LPS indicated as *^^^p* < 0.05; VENT vs. VENT + LPS indicated as *p < 0.05.

Plasma IL-6 levels in VENT + LPS fetuses were significantly higher compared to both UVC and VENT fetuses 12 h after starting ventilation (*p* = 0.014 and *p* = 0.017, respectively; Figure 3).

### 3.3 Effects of LPS and ventilation on gene expression

**Figure 4:**
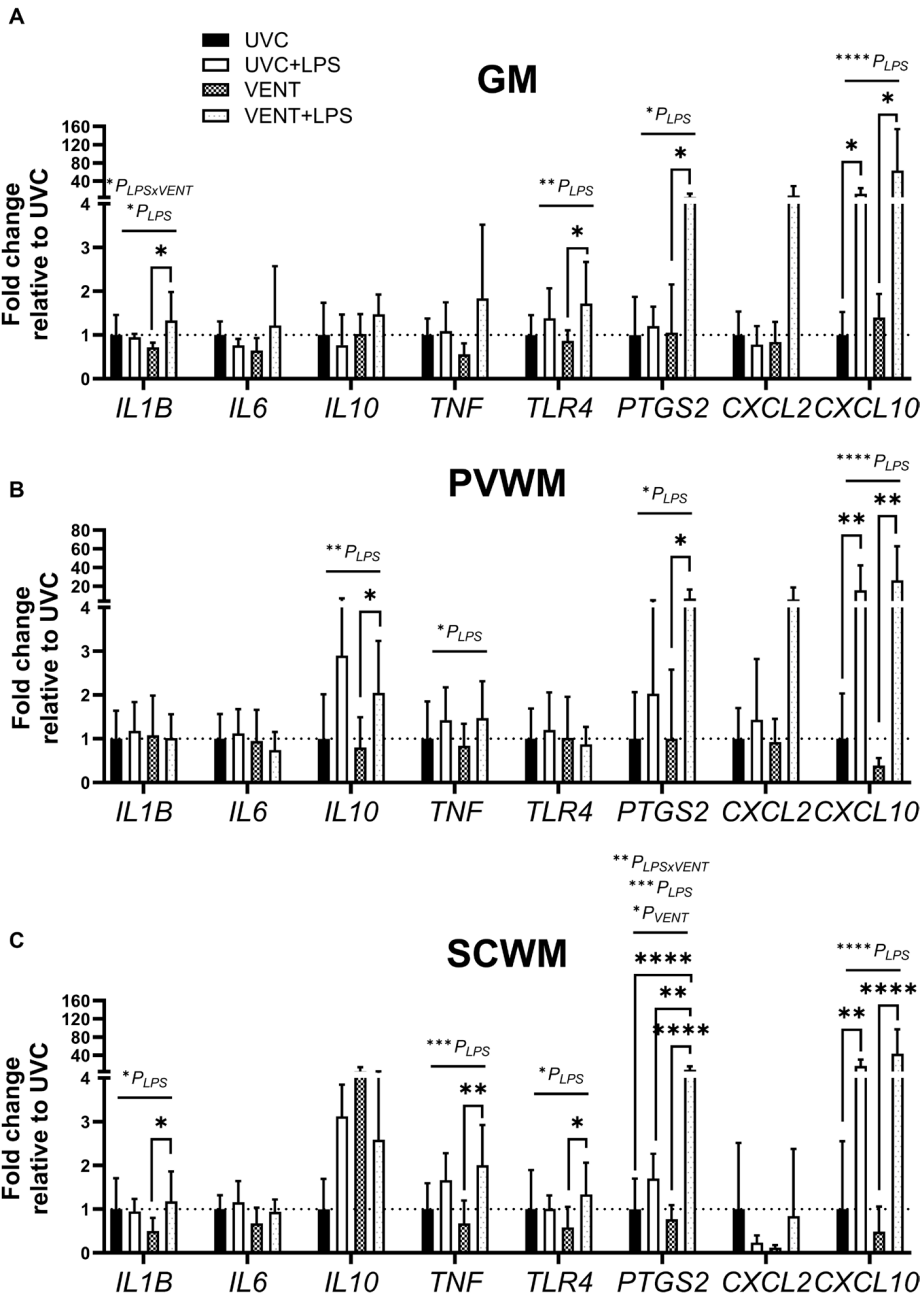
Fold change of mRNA expression of genes relating to inflammation. mRNA expression (relative to UVC) of genes relating to inflammation measured within **(A)** the cortical gray matter (GM), **(B)** periventricular white matter (PVWM) and **(C)** subcortical white matter (SCWM) in unventilated control (UVC; *n* = 7), LPS unventilated (UVC + LPS; *n* = 7), ventilated vehicle (VENT; *n* = 8), and ventilated LPS (VENT + LPS; *n* = 7) fetuses. Data are mean ± SD. Two-way ANOVA and Tukey’s multiple comparisons, *p < 0.05, **p < 0.01, ***p < 0.001, ****p < 0.0001.

The effect of LPS and *in utero* ventilation on gene expression in the cortical GM, PVWM and SCWM of the fetal sheep brain are summarized in Figure 4.

#### 3.3.1 Independent effects of ventilation and LPS on gene expression

LPS administration, irrespective of ventilation, resulted in up-regulation of mRNA expression of multiple genes involved in inflammation. Within the GM and SCWM, *IL1B* and *TLR4* were increased in LPS-exposed fetuses (all *p_LPS_* < 0.05; Figure 4). *TNF*, *CXCL10,* and *PTGS2* (the gene encoding cyclooxygenase 2 involved in producing prostaglandins) were also increased in LPS-exposed fetuses (all *p_LPS_* < 0.05 except *TNF* expression in the GM *p_LPS_* = 0.064; Figure 4). In the PVWM, the expression of *IL10* was increased in LPS-exposed fetuses (*p_LPS_* = 0.005; Figure 4). No significant effects of ventilation alone were found on mRNA levels of any genes assessed.

#### 3.3.2 Combined effects of LPS and ventilation on gene expression

In ventilated fetuses, exposure to LPS exacerbated increased expression of *IL1B, IL10, TNF, TLR4* and *PTGS2*, with expression higher in VENT + LPS fetuses compared to VENT only fetuses (all *p* < 0.05; Figure 4). *PTGS2* expression in the SCWM of VENT + LPS group was significantly higher compared to all other groups (vs. UVC, *p* < 0.0001; vs. UVC + LPS, *p* = 0.006; and vs. VENT, *p* < 0.0001; Figure 4).

### 3.4 Effects of LPS and ventilation on glial and neuronal cell density

**Figure 5:**
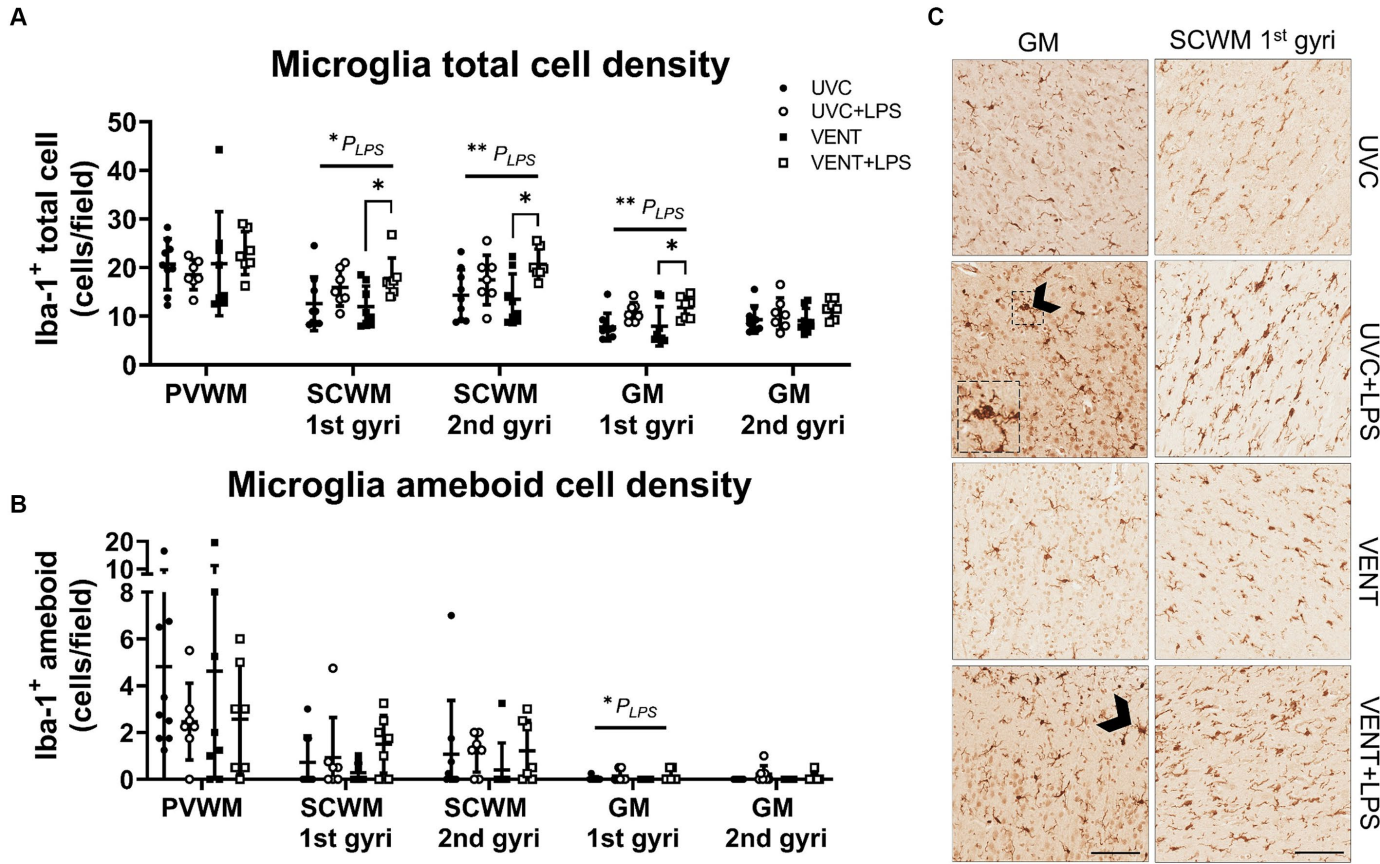
Iba-1 positive immunohistochemistry. **(A)** Iba-1 immunopositive cell density indicating microglia population and **(B)** ameboid or activated microglia cell density. Data are means ± SD. Two-way ANOVA and Tukey’s multiple comparisons, *p < 0.05. Unventilated control (UVC; *n* = 7), LPS unventilated (UVC + LPS; *n* = 7), ventilated vehicle (VENT; *n* = 8), and ventilated LPS (VENT + LPS; *n* = 7) fetuses. **(C)** Representative images of Iba-1-positive cells indicating microglia in the subcortical white matter (SCWM), cortical gray matter (GM), putamen and caudate. Arrowheads indicate ameboid microglia morphology. Insert are zoomed images of dashed box. Scale bar represents 100 μm.

In LPS-exposed fetuses, numbers of Iba-1-positive cells were increased in the SCWM (1st and 2nd gyri) and GM (1st gyri) (*p*_LPS_ = 0.010; *p*_LPS_ = 0.006; *p*_LPS_ = 0.003, respectively; Figures 5A,C). The increase in numbers of microglia in LPS-exposed fetuses was greatest in the VENT + LPS fetuses compared to VENT alone (SCWM 1st gyri: *p* = 0.021; SCWM 2nd gyri: *p* = 0.016 and GM 1st gyri: *p* = 0.037). In LPS-exposed fetuses, numbers of ameboid microglia were increased within the GM (1st gyri) (*p*_LPS_ = 0.044; Figures 5B,C).

Microglial aggregation area within the white and gray matter regions did not differ between groups (all *p* > 0.05; Supplementary Table 3).

**Figure 6:**
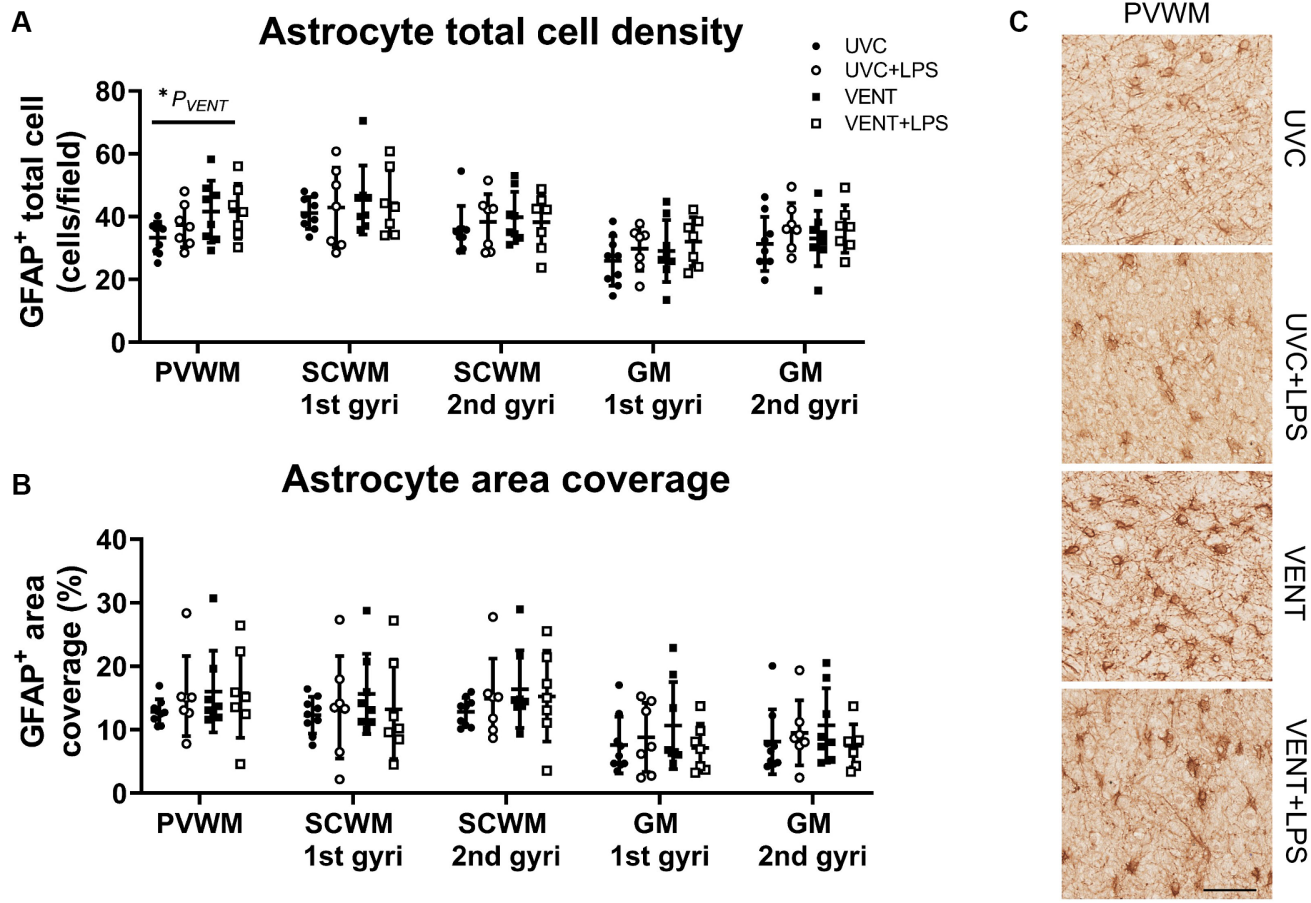
GFAP positive immunohistochemistry. **(A)** Numbers of GFAP immunopositive cells and **(B)** area coverage of GFAP staining. Data are mean ± SD. Two-way ANOVA and Tukey’s multiple comparisons, *p < 0.05. Unventilated control (UVC; *n* = 7), LPS unventilated (UVC + LPS; *n* = 7), ventilated vehicle (VENT; *n* = 8), and ventilated LPS (VENT + LPS; *n* = 7) fetuses. **(C)** Representative images of GFAP-positive cells. Scale bar represents 100 μm.

In ventilated fetuses, numbers of astrocytes were significantly increased in the PVWM (*p*_VENT_ = 0.03; Figure 6). No other changes to astrocyte cell density or area coverage were found.

**Figure 7:**
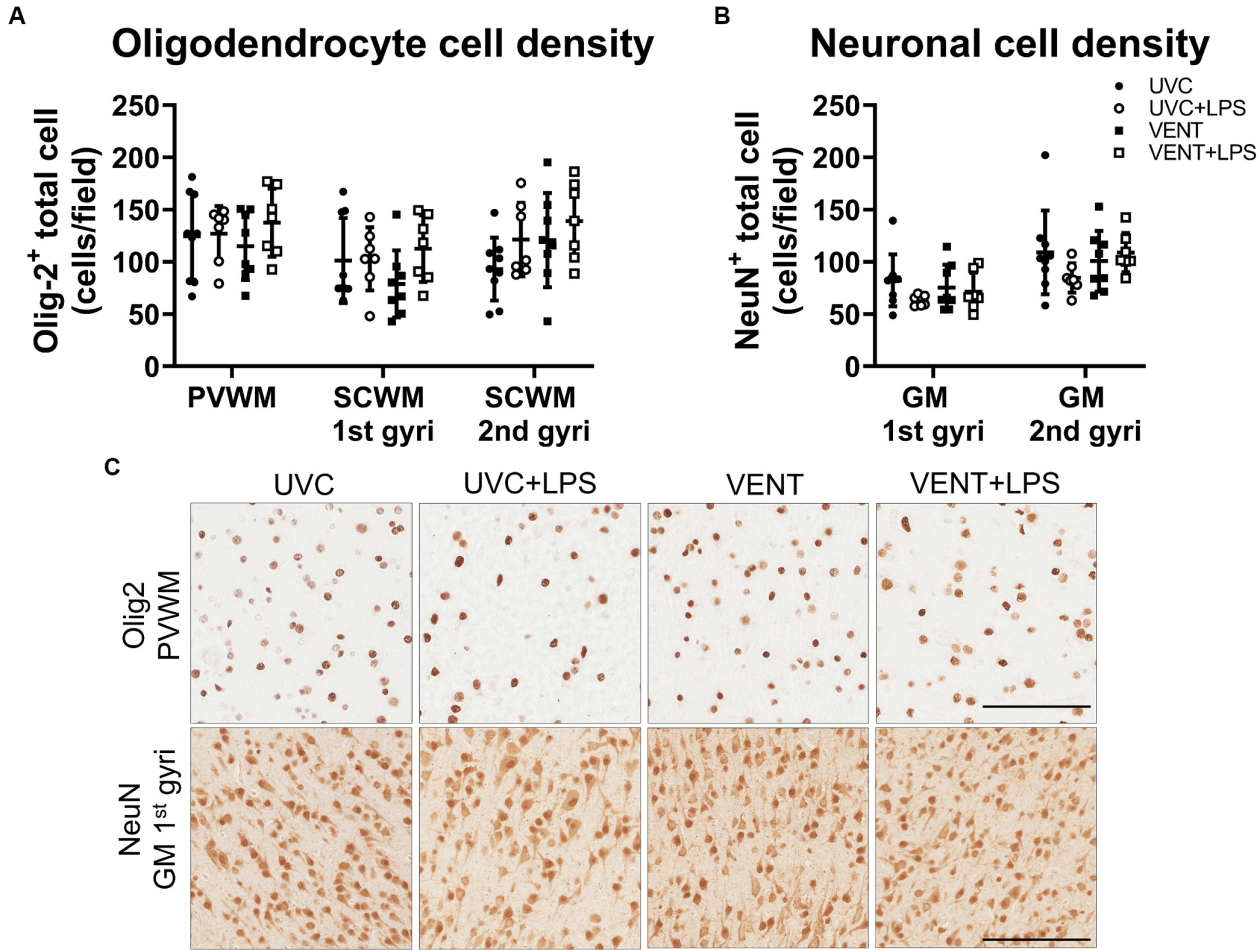
Olig2 and NeuN positive immunohistochemistry. **(A)** Numbers of Olig-2 and **(B)** NeuN immunopositive cells. Data are mean ± SD. Two-way ANOVA. Unventilated control (UVC; *n* = 7), LPS unventilated (UVC + LPS; *n* = 7), ventilated vehicle (VENT; *n* = 8), and ventilated LPS (VENT + LPS; *n* = 7) fetuses. **(C)** Representative images of Olig-2 and NeuN-positive staining in the periventricular white matter (PVWM) and cortical gray matter (GM). Scale bar represents 100 μm.

Numbers of Olig2-positive oligodendrocytes and NeuN positive neurons did not differ between groups for any of the white and gray matter regions examined (all *p* > 0.05; Figure 7).

## 4 Discussion

Inflammation is a key mechanism underlying the pathogenesis of perinatal brain injury. We investigated the relative contributions of intrauterine inflammation and subsequent mechanical ventilation, two of the most common causes of inflammation-induced perinatal brain injury, on markers of brain inflammation and injury in preterm fetal sheep. We show that 24 h of mechanical ventilation in of itself caused modest effects on markers of brain inflammation, whereas intratracheal LPS alone resulted in upregulation of pro-inflammatory related processes, which included increased microgliosis and increased mRNA expression of pro-inflammatory mediators in the cerebral white matter and gray matter. Importantly, combined exposure to LPS and mechanical ventilation amplified circulating levels of the pro-inflammatory cytokine IL-6, increased microglial density and amplified mRNA expression of pro-inflammatory markers. These observations suggest a synergistic inflammatory response of intrauterine inflammation and mechanical ventilation on the immature brain.

In this study, 24 h of *in utero* ventilation alone increased astrocyte cell density in the PVWM suggesting 24 h of mechanical ventilation had a modest effect on brain inflammatory markers. The increase in astrocyte cell density was associated with no change in astrocyte area coverage, potentially indicating a change in morphological state of these astrocytes with more retracted processes, indicative of an activated state (Schiweck et al., 2018). These findings are similar to those from a 24 h postnatal ventilation study, whereby no changes to numbers of microglia were observed in the white matter of late gestation lambs delivered at 125 dGA compared to non-ventilated lambs (Malhotra et al., 2018). However, in a separate study, mechanical ventilation of newborn lambs at a similar gestation (128 dGA) for 48 h resulted in significant gray and white matter gliosis and increased markers of neuronal and oligodendrocyte apoptosis (Nott et al., 2020). Potentially, the limited increase in markers of brain inflammation observed after 24 h of mechanical ventilation could be due to the limited duration of mechanical ventilation. Collectively, these data suggest that extending the duration of *in utero* mechanical ventilation to 48 h may promote a greater increase in markers of neuroinflammation and injury. Indeed, we have shown that 24 h of *in utero* ventilation, in the absence of LPS, increases inflammation in the lungs and caused a modest increase in inflammatory markers, with increased numbers of activated microglia but no differences in astrocyte immunoreactivity, in brainstem respiratory centers of preterm fetal sheep (Vidinopoulos et al., 2023; Azman et al., 2024). The difference in inflammatory presentation within the brainstem (increased respiratory center microgliosis) and cerebrum (increased periventricular astrocytosis) after mechanical ventilation could relate to how systemic inflammation is relayed to the central nervous system, which can occur across the blood brain barrier or chemosensory signaling via the brainstem dorsal vagal complex (Goehler et al., 2000; Marvel et al., 2004). For example, brainstem microglial activation has been linked to inflammation-induced activation of vagus nerve afferents (Quan and Banks, 2007; Pavlov and Tracey, 2012; Kaczmarczyk et al., 2017), whereas in the periventricular white matter, astrocytosis caused by endothelial activation at the endothelial-astrocyte interface could play a more prominent role in modulating the response to systemic inflammation (Galea, 2021). Furthermore, in this study, we achieved tidal volumes of 3–5 mL·kg^−1^ throughout the 24 h ventilation period. This relatively gentle ventilation strategy and the intact umbilical circulation would have limited the local inflammation and injury response within the brain, when compared to other studies investigating VIBI that delivered larger tidal volumes during mechanical ventilation (Bel et al., 1994). Furthermore, 24 h of mechanical ventilation had no effect on cerebral white and gray matter mRNA expression of inflammatory genes, which is consistent with previous studies showing no differences in brainstem inflammatory gene expression in the same experimental paradigm.

In the present study, we used intratracheal LPS administration to simulate intrauterine inflammation. Intratracheal LPS causes translocation of the endotoxins from the airspaces to the systemic circulation resulting in a systemic inflammatory cascade (Kramer et al., 2002; Polglase et al., 2009; Barton et al., 2015). Endotoxin exposure is first recognized by Toll-like receptor 4 (TLR4). This TLR4-dependent signaling leads to pulmonary and systemic cytokine release (e.g., IL-6, IL-1β and TNF) that can ultimately result in cerebral inflammation (Chen and Nuñez, 2010). Indeed, intratracheal LPS upregulated gene expression of pro-inflammatory cytokines *IL1B* and *TNF, TLR4*, prostaglandin-endoperoxide synthase (*PTGS2*), and pro-inflammatory chemokine *CXCL10*, and increased numbers of microglia in the white and gray matter after 24 h, which is consistent with the known neuroinflammatory response after LPS-exposure (Galinsky et al., 2020). However, when LPS-exposed fetuses were mechanically ventilated, markers of systemic and brain inflammation were exacerbated. We observed increased systemic IL-6 levels in VENT + LPS fetuses compared to both VENT and UVC groups along with greater upregulations of pro-inflammatory gene expression (specifically *TNF*, *TLR4* and *PTGS2*) and increased microglial density and activation in VENT + LPS fetuses compared to VENT fetuses. Collectively, these data suggest there was a synergistic effect of intrauterine inflammation and mechanical ventilation on markers of neuroinflammation in the preterm brain. Interestingly, *PTGS2* expression was particularly affected by the combination of LPS and ventilation. Increased *PTGS2* expression and subsequent prostaglandin E2 (PGE_2_) synthesis has been reported in the circulation and brain parenchyma of preterm humans and fetal sheep exposed to infection/inflammation (Westover et al., 2012; Siljehav et al., 2015; Stojanovska et al., 2022). In addition, increased PGE_2_ levels are associated with respiratory distress and the need for respiratory support (Clyman et al., 1980; Hofstetter et al., 2007). Prostaglandin synthesis is strongly associated with microglial activity (Monif et al., 2016). The increase in *PTGS2* expression is therefore consistent with the increase in microglial density in VENT + LPS fetuses compared to UVC and VENT groups.

*In utero* ventilation alone did not cause any changes to blood chemistry, while LPS exposure decreased pH, and PaO_2_, and increased PaCO_2_ and lactate concentration, consistent with previous studies (Dalitz et al., 2003; Galinsky et al., 2013, 2020). However, in fetuses ventilated after LPS exposure, there was a further decrease in pH and increase in PaCO_2_ and lactate compared to VENT and UVC fetuses. Indeed, systemic inflammation impairs placental function, increases tissue oxygen consumption and alters oxygen demand in fetal sheep (Dalitz et al., 2003; Galinsky et al., 2013, 2020). The combination of intrauterine inflammation and mechanical ventilation may have induced an even greater increase in tissue oxygen consumption and systemic metabolism akin to the increase in systemic inflammation observed in LPS + VENT group. These data support the synergistic effect of mechanical ventilation and intrauterine inflammation on systemic inflammation.

A limitation of this study is that assessments were conducted at a single time point (24 h). Whilst this provides important insight into the acute pathophysiological pathways of systemic and central nervous system inflammation, it does not fully reflect the dynamic and evolving nature of brain injury and repair. We observed no differences in total neuronal and oligodendrocyte numbers between the cohorts. The consequences of mechanical ventilation and/or LPS may be more evident with time or with longer durations of mechanical ventilation. For example, induction of the prostaglandins and increased circulating pro-inflammatory cytokines are associated with diffuse white matter injury and inhibition of neuronal and oligodendrocyte maturation (Shiow et al., 2017; Kelly et al., 2021, 2023). Thus, it is possible that this early inflammation has the potential to promote subsequent neurological injury that manifests beyond the first 24 h. Future assessments at a later timepoint are needed to confirm this. In addition, the fetuses in both LPS groups were older (118 dGA vs. 114 dGA) and heavier than the non-LPS fetuses. However, it is unlikely that the immune responses to ventilation or LPS-exposure would differ within this short period of gestational development (Park et al., 2020).

### 4.1 Clinical implications and considerations

The ventilatory strategy used in this study was one that aimed to simulate a gentle cardiorespiratory and cerebrovascular transition by using lower tidal volumes. While we did demonstrate that this ventilation strategy, in the absence of LPS, induces minimal neuroinflammation at 24 h, the inflammatory consequences following mechanical ventilation after intratracheal LPS exposure amplifies systemic and central nervous system pathways of neuroinflammation. We have shown previously that irrespective of the ventilatory strategy, continuous positive airway pressure (CPAP) or mechanical ventilation (PPV), prior inflammatory exposure resulted in similar degrees of inflammation within the lungs and the circulation (Polglase et al., 2009). Collectively, these data suggest that 24 h of mechanical ventilation *per se* may not be a major influence on neuroinflammation; but instead, what is critical is the degree of systemic and central nervous system inflammation at the time of birth. Overall, these data suggest that targeting systemic and central nervous system inflammatory pathways are likely to be key for improving interventions for preterm neuroprotection (Kelly et al., 2023).

## 5 Conclusion

In this study, we demonstrate that 24 h of mechanical ventilation in preterm fetal sheep has limited effects on markers of neuroinflammation or injury responses. However, exposure to LPS-induced systemic and central nervous system inflammation prior to 24 h of mechanical ventilation, augmented systemic and brain mRNA and histological markers of inflammation. These data suggest that exposure to intrauterine inflammation and subsequent mechanical ventilation could have a synergistic effect on neuroinflammation and injury in the preterm brain.

## Data availability statement

The original contributions presented in the study are included in the article/Supplementary material, further inquiries can be directed to the corresponding authors.

## Ethics statement

The animal study was approved by Hudson Institute of Medical Research Animal Ethics Committee. The study was conducted in accordance with the local legislation and institutional requirements.

## Author contributions

NT: Conceptualization, Data curation, Formal analysis, Investigation, Validation, Visualization, Writing – original draft, Writing – review & editing. AS: Data curation, Investigation, Visualization, Writing – review & editing. KV: Data curation, Investigation, Visualization, Writing – review & editing. ZA: Data curation, Investigation, Visualization, Writing – review & editing. YP: Data curation, Investigation, Methodology, Visualization, Writing – review & editing. VZ: Data curation, Investigation, Writing – review & editing. KCh: Data curation, Investigation, Visualization, Writing – review & editing. SH: Conceptualization, Methodology, Resources, Software, Writing – review & editing. KCr: Conceptualization, Methodology, Resources, Writing – review & editing. BA: Conceptualization, Data curation, Investigation, Methodology, Project administration, Resources, Software, Supervision, Writing – review & editing. RG: Conceptualization, Data curation, Formal analysis, Funding acquisition, Investigation, Methodology, Project administration, Resources, Software, Supervision, Visualization, Writing – original draft, Writing – review & editing. GP: Conceptualization, Data curation, Formal analysis, Funding acquisition, Investigation, Methodology, Project administration, Resources, Software, Supervision, Visualization, Writing – original draft, Writing – review & editing.
