# Supplementary material for: The synergistic effects of mechanical ventilation and intrauterine inflammation on cerebral inflammation in preterm fetal sheep
Source: Front Cell Neurosci. 2024 Jun 19;18:1397658. doi: 10.3389/fncel.2024.1397658 (PMC11220153; doi:10.3389/fncel.2024.1397658)
Supplement: Supplementary file 1 [file Table_1.DOCX]

Supplementary Material

# Supplementary Data

ARRIVE Guidelines. See Supplementary Data file 1

# Supplementary Tables

## Supplementary Table 1

|  | **UVC** | **UVC+LPS** | **VENT** | **VENT+LPS** |  |  |  |
| --- | --- | --- | --- | --- | --- | --- | --- |
| Number (n) | 7 | 7 | 8 | 7 | ***Statistics*** | | |
| Male:Female | 3:4 | 2:5 | 4:4 | 3:4 | *p***_VENT_** | *p***_LPS_** | *p***_LPS X VENT_** |
| Gestational age (d) | 114 ± 2 | 118 ± 0 | 114 ± 2 | 118 ± 0 | 0.733 | **<0.0001*** | 0.733 |
| Body weight (kg) | 2.4 ± 0.4 | 2.7 ± 0.2 | 2.5 ± 0.4 | 2.8 ± 0.4 | 0.548 | **0.018*** | 0.735 |
| Brain weight (g) | 36.1 ± 3.6 | 39.3 ± 2.1 | 35.6 ± 2.6 | 36.6 ± 2.5 | 0.156 | 0.069 | 0.316 |
| Lung liquid volume (mL) | - | - | 111.3 ± 30.9 | 132.9 ± 24.3 | - | - | - |
| Ventilation duration (h) | - | - | 24.1 ± 0.2 | 23.9 ± 0.4 | - | - | - |

**Animal characteristics at post-mortem and ventilation-related parameters.** Data expressed as mean ± SD and analysed by Two-Way ANOVA and Mann-Whitney test. Significance was set at **p*<0.05.

## Supplementary Table 2

|  | **Statistics** | | | | | | |
| --- | --- | --- | --- | --- | --- | --- | --- |
|  | **Main Effects** | | | **Interactions** | | | |
|  | *p*_LPS_ | *p*_VENT_ | *p*_TIME_ | *p_LPS X TIME_* | *p_VENT X TIME_* | *p_LPS X VENT_* | *p_LPS X VENT X TIME_* |
| pH | **0.026*** | 0.103 | **<0.0001****** | **0.002**** | 0.780 | **0.016*** | **<0.0001****** |
| PaCO_2_ | **0.001**** | **0.037*** | **<0.0001****** | **0.004**** | 0.567 | 0.168 | 0.159 |
| PaO_2_ | **0.004**** | **0.037*** | **<0.0001****** | **<0.0001****** | 0.348 | 0.298 | 0.185 |
| Glucose | 0.538 | 0.471 | **0.049*** | **0.016*** | **0.0001***** | 0.715 | 0.391 |
| Lactate | **0.006**** | 0.130 | **<0.0001****** | **<0.0001****** | 0.131 | 0.731 | 0.257 |

Summary of the overall interactions between LPS (*p*_LPS_) and ventilation (*p*_VENT_) across the experiment time (*p*_TIME_) obtained from unventilated control (UVC; n=7), LPS unventilated (UVC+LPS; n=7), ventilated (VENT; n=8), and ventilated LPS (VENT+LPS; n=7) fetuses for blood gas and metabolite and plasma IL-6 concentrations. Data are statistical output analysed by Three-Way ANOVA. Significant effects are bolded and **p*<0.05, ***p*< 0.01, ****p*< 0.001, *****p*< 0.0001.

## Supplementary Table 3

| **Staining** | **Brain Region** | **Group** | | | | **Statistics** | | |
| --- | --- | --- | --- | --- | --- | --- | --- | --- |
|  |  | **UVC** | **UVC+LPS** | **VENT** | **VENT+LPS** | *p*_VENT_ | *p*_LPS_ | *p_LPS X VENT_* |
| **% of brain region area covered by microglial aggregations (%)** | SCWM | 4.77 ± 7.04 | 4.69 ± 4.07 | 2.31 ± 5.20 | 1.95 ± 2.49 | 0.174 | 0.908 | 0.941 |
|  | GM | 0.00 ± 0.00 | 0.00 ± 0.00 | 0.00 ± 0.00 | 0.00 ± 0.00 | - | - | - |
|  | PVWM | 13.24 ± 20.28 | 8.21 ± 6.75 | 3.04 ± 3.67 | 2.56 ± 2.46 | 0.058 | 0.496 | 0.574 |

**Effects of LPS and ventilation on microglial aggregations.** Microglial aggregations assessed from Iba-1 positive immunostaining in unventilated control (UVC; n=7), LPS unventilated (UVC+LPS; n=7), ventilated (VENT; n=8), and ventilated LPS (VENT+LPS; n=7) fetuses. All data are mean ± SD and analysed by two-way ANOVA. Subcortical white matter (SCWM); cortical grey matter (GM); periventricular white matter (PVWM).
